# The organizational digital shadow of the certified cancer center—from yearly snapshots to continuous signals

**DOI:** 10.3389/fdgth.2026.1955074

**Published:** 2026-10-21

**Authors:** Markus Knott, Markus Krebs

**Affiliations:** 1 Stuttgart Cancer Center (SCC)—Tumorzentrum Eva Mayr-Stihl, Klinikum Stuttgart, Stuttgart, Germany; 2 Comprehensive Cancer Center Augsburg, University Medical Center Augsburg and Medical Faculty, University of Augsburg, Augsburg, Germany; 3 Bavarian Cancer Research Center (BZKF), Augsburg, Germany

**Keywords:** certified cancer center, continuous auditing, digital shadow, digital twin of an organization, governance, health informatics, oncology, quality assurance

## Abstract

Certified cancer centers produce quality continuously but measure it discretely: Conformity is established in a periodic audit that samples a year of institutional life in 1 or 2 days, and demonstrating it costs clinician time. However, the same institution continuously emits data traces—indicator sheets, tumor board minutes, audit findings, registry submissions, trial management records—that differ in structure and in latency but already exist. We argue that they constitute an organizational digital shadow: a unidirectional, purpose-bound, deliberately incomplete projection of institutional conformity, refreshed at whatever latency each source permits and read against a versioned certification standard. We choose shadow deliberately. Organization scholars and process-mining researchers question whether a faithful, bidirectional twin of an organization is achievable at all, while engineering research distinguishes digital models, shadows, and twins by their degree of data flow integration. Applied to the certified cancer center, that taxonomy yields an actionable claim: What is technically within reach today is a shadow, and we argue that a shadow could suffice to turn quality assurance from a periodic verdict into a continuous signal. We connect three bodies of literature that address patient physiology, hospital throughput, or financial control rather than institutional conformity and propose a layered architecture, a maturity progression separating technical coupling from analytical function, and a governance analysis. Because automation earns legitimacy by relieving staff, the byproduct principle is a design constraint. Certified centers are a tractable first instantiation: Few organizations possess both a normative standard and a structured data stream to project against it.

## 1 Introduction: the audit paradox

A certified cancer center in the German system is audited annually. Over 1 or 2 days, auditors review the updated indicator sheet, sample tumor board documentation, interview staff, and inspect corrective actions. The certificate is valid for 3 years, with surveillance audits in between and a full recertification audit at the end; centers with consistently strong results may have on-site surveillance replaced by document review. Conformity with several hundred requirements is therefore established from a sample taken on 1 or 2 days a year, sometimes from documents alone.

The procedure is not defective. The certification program of the German Cancer Society (DKG), operated through its institute OnkoZert, has professionalized cancer care by placing multidisciplinary teams at the center of the pathway (1). It is now the largest oncological certification system in Europe: Centers in Austria, Switzerland, Italy, and Luxembourg have participated since 2014, and the European Cancer Centre Certification Programme, launched in 2016, opened it to non-German-speaking countries (1, 2). Its effect is measurable, and the pattern is instructive. In registry data on 47,440 patients, the adjusted hazard ratio for death was 0.92 (95% CI 0.89–0.96) for colon cancer, with no significant difference in rectal cancer overall but a significant benefit in curatively treatable UICC I–III disease (3); in 160,935 insured patients, the ratio was 0.92 for both entities (4). Certification works most visibly where guideline adherence is most tightly specified.

But the procedure embodies a structural asymmetry: Quality is produced continuously and measured discretely. A tumor board may drift out of conformity in February because a required discipline is no longer reliably represented, or because its case volume is on course to miss the annual minimum. The non-conformity, however, is detected only in November. In between, patients are treated in a configuration that no longer matches the one the certificate attests to. Whether that means worse outcomes is unknown—which is the point: Nobody is measuring.

Accounting encountered this asymmetry decades ago. Because traditional audit is periodic, evidence may be gathered long after the events it concerns; that observation motivated continuous auditing from the early 1990s (5), which replaced sampling with automated testing against rules and periodic verdicts with ongoing assurance (6).

The asymmetry has a second face. Demonstrating conformity is expensive in the currency oncology can least afford. In one certified center, redesigning the documentation workflow of 12 tumor boards cut coordinators’ preparation time from 66.0 to 46.5 h per week and the preparation workload of radiologists and pathologists by 22.9% and 52.7%, respectively (7). That study addressed one data-dense process; in our experience as certification auditors and center leads in university and large municipal cancer care providers, the pattern recurs across the certification apparatus, with staff time spent re-entering evidence that already exists in the institution's systems. We offer this as practice-based observation, not measured result. It sets the design constraint: Any proposal to measure more often must replace documentation work, not add to it.

Healthcare has not made this transition, but it has quietly acquired the raw material. The trail a certified center already emits, largely as a byproduct of certification, documentation, and reimbursement, is an exploitable substrate. We name it the organizational digital shadow and argue that naming it correctly is the precondition for building it responsibly.

## 2 Conceptual foundations: digital model, digital shadow, and digital twin

The term digital twin has become semantically overloaded. Engineering research addressed this early: A widely accepted classification distinguishes three levels by the degree of automated data flow integration between physical and digital object (8) (Figure 1).

- Digital Model—no automated exchange in either direction; changes do not propagate.
- Digital Shadow—an automated one-way flow from physical to digital; the digital state follows, not the reverse.
- Digital Twin—an automated bidirectional flow; the digital object can act back upon the physical oneDigital Shadow—an automated one-way flow from physical to digital; the digital state follows, not the reverse.

**Figure 1:**
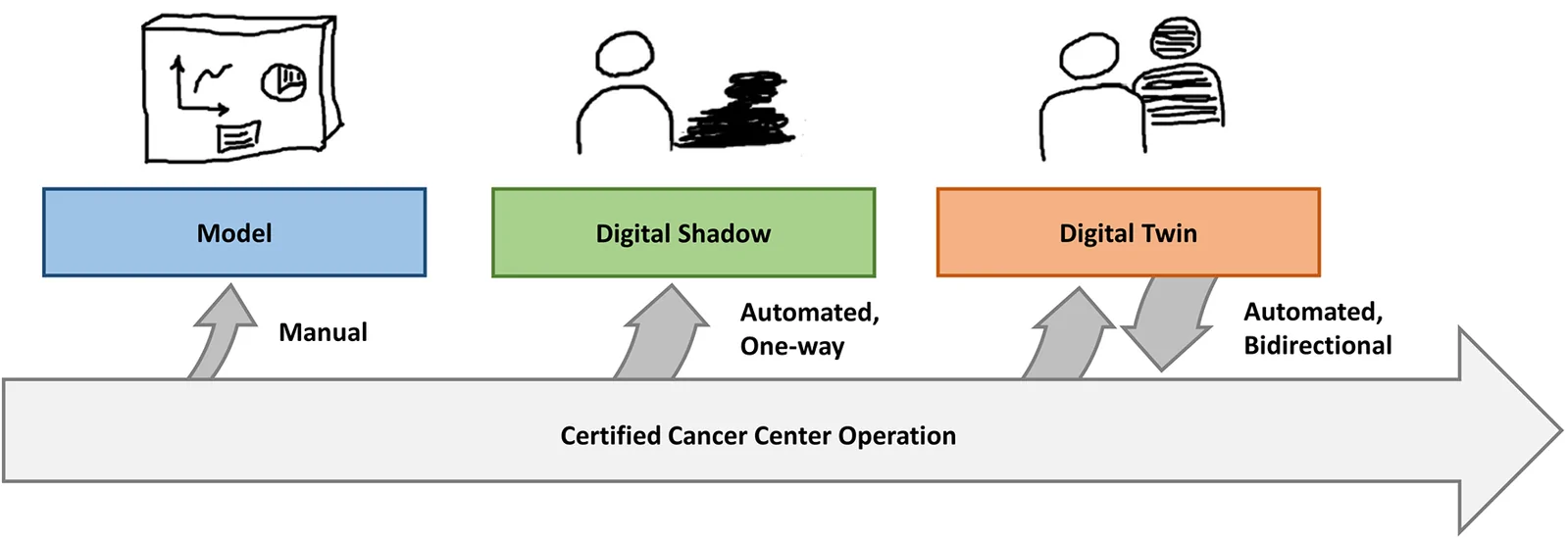
Three levels of data flow integration between an institution and its digital representation, following the classification of Kritzinger et al. (8). In the digital model (labeled “Model” in the figure), data are transferred manually and changes do not propagate on their own; in the digital shadow, an automated one-way flow runs from the institution to its representation; in the digital twin, the flow is automated and bidirectional, so that the representation can act back upon the institution. The reference object throughout is the operation of the certified center rather than an individual care pathway; this is the transfer proposed here, whereas the existing healthcare literature applies the same three levels at the patient and process levels (Section 4). Original figure; the level definitions follow (8).

We adopt this taxonomy as an explicit operational convention rather than a universally binding definition. ISO/IEC 30173:2023 defines a digital twin more broadly—by data connections that enable convergence between physical and digital states at an appropriate rate of synchronization—and treats the control loop as a separate concept (9); bidirectionality is not a defining criterion there, and an organizational digital shadow (ODS) could fall within that broader term. We keep the stricter convention because the line it draws—whether the representation can act back upon the institution—is the one that carries accountability weight in a clinical organization (Section 5). Judged by its criterion, only 18% of the systems described as digital twins in a survey of the manufacturing literature implemented the bidirectional data flow the term requires; the remaining systems were digital shadows, mere digital models, or not classifiable (8). The same inflation is under way in healthcare (10).

A sustained engineering program has since treated the shadow as an end state rather than a way station (11, 12). Its argument is instructive: In systems of realistic complexity, twins covering more than a few perspectives are neither computationally feasible nor especially useful, whereas shadows, being compact purpose-driven views that combine condensed measurement data with simplified models, deliver more practical value at a fraction of the cost (11, 12). Formally, such a shadow generalizes the database notion of a view (13).

A digital twin makes an epistemic promise, fidelity, and an operational one, control. In a certified cancer center, the first cannot be fully kept and the second should not be made lightly.

## 3 The organization as object: what exists outside healthcare

A common assumption in health informatics is that digital twins are patient- or device-level constructs and the organizational level is left unexplored. This is inaccurate.

### 3.1 The digital twin of an organization is an established concept

Since 2017, the digital twin of an organization (DTO) has described a dynamic software model that uses operational and contextual data to represent how an organization deploys resources and delivers value. Parmar, Leiponen, and Thomas stress that it emerges through incremental construction rather than a single build (14).

### 3.2 Process mining supplied the concrete realization

Park and van der Aalst implemented a DTO using action-oriented process mining, in which a digital twin interface model represents processes as object-centric Petri nets, a notation in which one model tracks several interacting object types at once, such as a patient, a specimen, and a board decision. Event data are continuously confronted with that model, violations evaluated and actions triggered (15). This is the closest existing analog to our proposal: a formal representation of what should happen, compared continuously against what did happen, with deviations surfaced as actionable findings.

### 3.3 The field has criticized itself, and that criticism is our foundation

Becker and Pentland ask whether a digital twin of an organization can be taken seriously, and answer that organizational routines have both an ostensive aspect, the shared and partly tacit understanding of the routine, and a performative aspect, the actions actually taken (16, 17). Event data capture only the latter. Lyytinen and colleagues add that a genuine twin presupposes a synchronized, two-way causal coupling between world and representation, and that fully modeling an organization would require modeling agency, conflict, and emergence (18). A socio-technical agenda frames the DTO as an aspiration surrounded by unresolved challenges (19).

The obstacles they name—unbounded organizational boundaries, shifting human behavior, and an unobservable ostensive layer—are not implementation gaps to be closed by better engineering. They are, on that analysis, properties of organizations—which questions the feasibility of a fully faithful organizational twin without establishing its impossibility. Hence:

Treating the organization as the object of digital representation is not novel. The twin framing at the organizational level is contested by its own proponents. The shadow framing is therefore not a lesser version of the twin; it is the correct one, and it has been available in the engineering literature all along.

## 4 What healthcare has built—and what it has not

Digital twin research in healthcare clusters into three well-populated strands: patient-level physiological twins, device-level twins, and, increasingly, hospital operations twins. The last is now real practice: simulation-based twins model patient flow, bed allocation, and capacity to reduce bottlenecks (20). In our own prior work, we introduced the health digital shadow alongside the health digital twin for modeling an individual patient's trajectory (21). The present paper carries that distinction to the institutional level, where the case for the weaker term is stronger still.

Systematic reviews report that clinical integration remains rare and the system level under-represented (22), while radiology-focused work identifies the departmental level as the least mature (23). The gap, stated precisely, is this:

Organizational digital twins in healthcare model throughput, resource allocation, and safety. Among the literature considered for this Perspective, which was not a systematic search, we identified none that evaluates an institution against a certification standard.

Such twins answer how many beds will be needed and where the bottleneck is. They do not answer whether this institution is, today, in conformity with the requirements that make it a certified cancer center and if not, since when and in which dimension. Conformity is a deliberately constructed proxy for the structural and procedural preconditions of good outcomes (1, 3); asking about it continuously rather than annually is the transition continuous auditing made in a different domain (5, 6). An institution can remain formally conformant while experiencing quality problems the requirements do not represent; the ODS inherits that limit from the standard it reads.

The only application of digital shadow inside an oncological institution that we found stops short of the organizational level: Cai and colleagues coupled a laboratory-information-system-based digital shadow with a Lean Six Sigma intervention in a tertiary cancer hospital, reducing median intra-laboratory turnaround time from 77.2 to 69.0 min (−10.6%, *p* = 0.0182) (24). That is proof of principle for one process; our proposal generalizes it to the certified quality system as a whole. A second, unrelated use of the term—a soft sensor forecasting CAR-T cell concentration in a perfusion bioreactor to time the harvest (25)—confirms that, in biomedicine, digital shadow is currently a process-engineering term.

### 4.1 What the ODS is not

Three neighboring instruments already extract quality signals from routine data. Electronic clinical quality measures compute patient-level indicators from electronic health record data for external reporting; even there, extraction remains hard, with coded data missing or miscategorized across most systems in one multipractice study (26). Quality dashboards visualize such indicators but leave the judgment to the reader. Conformance checking in process mining compares event data against a process model (15). The ODS differs in reference object rather than technique: It evaluates an institution against a versioned certification standard spanning structure, process, outcome, and governance together and returns a conformity finding with provenance and time to detection rather than a chart. Two features carry the difference from the digital twin of an organization (15). The reference model is not a process model the institution specifies for itself but a standard maintained, versioned, and enforced by an external body, to which the institution has committed (1); versioning matters because a changed result may reflect a revised requirement rather than a change in practice, so each finding carries the requirement version it was judged against. In addition, the reflexive layer, which records how detected deviations are handled, is not a first-class object in the DTO, whose action step the ODS deliberately omits. Its structural and governance layers have no direct counterpart in any of the three.

## 5 The organizational digital shadow of a certified cancer center

We define the organizational digital shadow (ODS) of a certified cancer center as follows:

A unidirectional, purpose-bound projection of an institution's conformity—the certification system's constructed proxy for care quality (1, 3)—assembled from the data traces it already generates in certified operation, refreshed at whatever latency each source permits, and evaluated against the versioned certification standard to which it has committed.

Three properties are load-bearing:

- *Unidirectional*: The shadow observes; it does not act. Actuation remains with clinicians and managers.
- *Purpose-bound*: It is defined by a question, such as whether the tumor board is in conformity, not by an ambition to represent the institution exhaustively (12, 13).
- *Standard-referenced*: Unlike a factory or a supply chain, a certified cancer center possesses an explicit, externally maintained normative model of itself: the certification requirements and indicator sheets (1). That makes it a particularly tractable first use case.

### 5.1 A layered architecture

We propose four layers, mapped onto Donabedian's structure–process–outcome framework (27) and extended by a reflexive governance layer (Table 1A). “Continuous” is accordingly latency-aware rather than real-time: Each layer is refreshed at the rhythm its sources permit, and a finding carries the age of the data it rests on.

**Table 1:** The organizational digital shadow.

**(A)** Layered architecture.			
Layer	Representative data traces	Donabedian level	Update rhythm
L1 — Structure	Personnel qualifications and continuing education records; device and infrastructure registers; cooperation agreements; network partner status	Structure	Event-driven (months)
L2 — Process	Tumor board documentation (case volume, pretherapeutic presentation rate, recommendation completeness, implementation rate); pathway timestamps (diagnosis → board → therapy); trial management screening and enrollment records	Process	Continuous (days)
L3 — Outcome	Cancer registry submissions; survival and recurrence data; complication and morbidity indicators; patient-reported outcomes	Outcome	Delayed (months–years)
L4 — Reflexive/governance	Audit findings; corrective and preventive actions; critical incident (CIRS) reports; complaints; internal audit minutes; indicator sheet submissions	(Meta-level)	Annual → continuous

**(B)** Maturity on two axes.				
Level	Technical coupling	Analytical function	Question it answers	Status
ODS-0	Manual (digital model)	Descriptive	What were our indicator values last year?	Current: observed state in most centers
ODS-1	Automated, one-way (digital shadow)	Descriptive	What are our indicator values now?	Technically within reach; integration work; not yet demonstrated
ODS-2	Automated, one-way (digital shadow)	Conformity-evaluating	Are we in conformity—and if not, since when and in which dimension?	Proposed here; needs machine-readable indicator definitions; not implemented
ODS-3	Automated, one-way (digital shadow)	Predictive	Will we still be in conformity at the end of the cycle?	Future; conditional on ODS-2 and validated models
—	Automated, bidirectional (digital twin)	Prescriptive/actuating	(not asked)	Deliberately out of scope

(A) Layered architecture, mapped to Donabedian levels. (B) Maturity on two orthogonal axes: technical coupling against analytical function; the Status column distinguishes current, proposed, and future capability. All ODS levels occupy the manual and one-way rows; the twin boundary is crossed only by automated actuation.

Layer 4 is the layer least discussed in the literature we considered and the one we find most interesting: the institution's record of how it responds to its own detected deviations. A shadow including L4 records not only conformity but also the institution's response to its own deviations—a proxy for, not proof of, the capacity to self-correct—closer to a dynamic capability (28) and double-loop learning (29) than to a key performance indicator. Whether a recorded correction also revised the assumptions behind the indicator—the double loop—is not something the record can show; that judgment stays with human review.

### 5.2 Management theoretic grounding

The ODS instantiates three management ideas that, to our knowledge, have not had a data substrate in oncological quality management (Supplementary Table S1):

- *Continuous auditing* (5, 6) supplies the operating principle: rule-based testing against all events instead of sampling, and ongoing assurance instead of a periodic verdict. The requirement catalogue plays the role internal control assertions play in finance.
- *Requisite variety* (30) and the *Viable System Model* (31) explain why selective modeling is unavoidable: A regulator can control only as much variety as it commands, and no representation commands the variety of the organization it stands for, so every representation attenuates that variety by selection. What follows is not that a shadow beats a twin, but that any organizational representation must be radically partial, and shadow is the word that says so.
- *Levers of control* (32) names the design risk: A continuously monitored institution can slide into purely diagnostic control, surveillance by dashboard. It earns its value only if also used interactively, as a shared object of managerial dialogue.

### 5.3 Where a center stands, and what its next step is

Because such a shadow is built incrementally rather than commissioned (14), centers need a way to locate themselves. Two dimensions must be kept apart, and the twin literature conflates them: technical coupling and analytical function (Table 1B). All four ODS levels sit in the one-way column—forecasting changes the analytical function, not the coupling, and does not move a shadow closer to a twin. Only automated actuation does that. ODS-2, the step this paper proposes, is where the normative reference enters: Event data are continuously confronted with the certification requirement model, and deviations surfaced with provenance and time to detection. None of the three ODS levels has been demonstrated in a certified center; their order here is the order in which we propose to build and test them.

### 5.4 A worked example

Take indicator 2a of the German Colorectal Cancer Center Programme, pretherapeutic case presentation: Elective patients with rectal cancer and elective patients with stage IV colon cancer are to be presented to the interdisciplinary tumor board before therapy begins, with a target of at least 95% (33). The chain that ODS-2 proposes runs as follows: the requirement, cited with the version of the indicator sheet; a rule—for every case in the denominator with a first-diagnosis date in the period, a board recommendation must precede the first therapy timestamp, and the period rate must not fall below 95%; source data—diagnosis date and stage from tumor documentation, recommendation and attendance from board minutes, therapy start from order entry or the operation record; evaluation—each case classified as conformant, non-conformant or unassessable; provenance—case and source-record identifiers, rule and standard versions, evaluation time; an alert—a time-stamped finding when the rate falls below 95% or the unassessable share exceeds its limit; and human review—the quality office confirms the finding, annotates a justified exception such as a malignancy recognized only at operation, or opens a corrective action, recorded in Layer 4. A dashboard shows the rate; the ODS returns each classification with its evidence and what was done about it.

We stop deliberately at ODS-3. Bidirectionality in a clinical organization is not primarily a technical step but one in which accountability for care decisions migrates toward a model. The loop nevertheless closes, through people: Alarms, management decisions, and corrective actions form a feedback path that is socio-technical rather than technical. Holding those two levels apart is the substance of refusing the word twin. The shadow is unidirectional where the data flow is, and responsibility for acting on what it shows stays where it belongs.

Three near-term steps follow: machine-readable formalization of the certification indicator definitions by the certifying bodies; a single-center ODS-2 demonstrator for the tumor board domain, using conformance checking from process mining (15); and a multicenter concordance study against conventional audit, with staff time as a coprimary endpoint.

## 6 Governance and regulatory considerations

The regulatory instruments cited here are primary legal sources; a peer-reviewed analysis of the device-versus-management-software boundary in European healthcare is drawn on in the following (34).

### 6.1 Medical Device Regulation (MDR)

Qualification turns on intended purpose. On the purpose defined here—observing organizational conformity, with no claim about an individual and no input into individual clinical decisions—the ODS would be a management instrument outside the MDR's device definition (35). Commission guidance confirms that software processing health information falls under the MDR only where it directly serves a medical purpose (36). That boundary is only as stable as the system's scope, and must therefore be a design constraint rather than an afterthought.

### 6.2 EU AI Act

Where the ODS employs AI components, for example, large language models extracting conformity signals from free-text tumor board minutes—an approach we previously proposed (34)—classification depends on use. On that scope, a system evaluating institutional rather than individual conformity appears unlikely to constitute a high-risk system under Annex III (37); classification attaches to a specified implementation, not to a concept. Two adjacencies warrant scrutiny: employment-related use, if deviations are attributed to individual clinicians, and drift toward clinical decision support. We treat transparency, human oversight, and documented provenance as design commitments rather than as obligations flowing from any classification.

### 6.3 Data protection and the European Health Data Space

The ODS would operate almost exclusively on data that already exist for treatment, documentation, quality assurance, and registry reporting, so a legal basis for the underlying processing would usually exist. Instead, purpose and aggregation change, and both require case-specific assessment under Articles 6 and 9 GDPR. The European Health Data Space establishes permit and secure-processing mechanisms for secondary use rather than a general license (38).

### 6.4 The soft-governance problem

The hardest question is not legal. Goodhart's law, that a measure ceases to be a good measure once it becomes a target (39), operates with far greater force under continuous than under annual measurement. Evidence from the English health service supports this: Real-time transparency of the target regime appeared to exacerbate reactive gaming rather than deter it, and the authors warn that surveillance on readily available data, substituted for site visits, widens rather than narrows the audit hole (40). An institution audited once a year can game 1 week; one shadowed continuously can be gamed continuously, and the gaming will look like conformity. Continuous observation improves detection when the deviation is unintended—a discipline that stops attending, a case volume that drifts—rather than chosen, when the indicator is a byproduct of data whose primary use is treatment or reimbursement, and when the finding is used interactively rather than as a target with consequences attached (32); where those conditions fail, improved indicators can coexist with unchanged practice. Provenance and gaming resistance are therefore first-class design requirements, and the human audit function must be retained to see what the shadow cannot (16). The reflexive layer is where gaming becomes visible: It looks like conformity in Layers 1–3 but leaves a trace in Layer 4—alerts dismissed without review, exceptions that accumulate—which is why that layer is read by the human auditor, not only by the shadow.

## 7 Limitations

### 7.1 Implementation

The architecture has not been implemented in a certified cancer center. Technical feasibility beyond ODS-1, organizational acceptance, cost, false-alert burden, and the effect on staff workload are therefore empirical questions; the near-term steps in Section 5 are the order in which we propose to answer them.

### 7.2 Interoperability

The data traces of a certified center live in mutually unaware systems: hospital, laboratory and imaging information systems, tumor documentation, trial management, and registry interfaces. Assembling a shadow means solving a semantic integration problem that healthcare has not solved elsewhere. Standards such as HL7 FHIR are necessary but not sufficient; the certification indicator definitions themselves would need machine-readable representation—arguably the most tractable next step, and one within reach of the certifying bodies.

### 7.3 Validation

An ODS makes claims about institutional conformity, and those must be validated. The annual audit is the only available comparator, and an imperfect one: itself sample-based and interpretive. The obvious design—running an ODS in parallel with conventional audits and measuring concordance, time to detection, and false-positive rate—is feasible and, to our knowledge, has not been done.

### 7.4 The ostensive residue

No shadow captures the unwritten. Some of the most consequential determinants of quality, such as whether a junior clinician feels able to dissent in a tumor board, leave no data trace. On the argument of other authors (16, 18), this is not a temporary limitation to be closed by better sensors. It is why the human auditor does not become obsolete, and why shadow is the honest word.

### 7.5 Burden and legitimacy

A quality instrument that increases documentation burden destroys what it measures. The byproduct principle is a constraint, not a convenience: The ODS is worth building only if it removes work currently done by hand. The relief is of one kind: Audit work consists of assembling and screening evidence and of interpreting it, and the ODS addresses only the first. Assembling attendance records and flagging gaps can be automated; whether a gap is an incomplete entry, a breached requirement, or a justified exception requires contextual judgment, which stays with the human auditor (16). Whether the net effect is relief remains an empirical question, to be measured against implementation, maintenance, and alert review. Manual compilation of indicator sheets before an audit is the clearest example; at ODS-1, it becomes a byproduct of systems already running. A component requiring new documentation should be rejected by default.

## 8 Conclusion

The certified cancer center is one of very few organizations already possessing both a formal, externally maintained model of what it ought to be and a continuous, structured record of what it actually did. It has the raw material to cast an organizational digital shadow—though not yet the integration, the machine-readable rules, or the validation that casting it would require—and uses that material for one annual snapshot.

We do not propose a digital twin of the cancer center. The management and information systems bodies of literature have argued on structural grounds that a faithful twin of an organization is at best a distant aspiration and possibly unattainable in principle (16, 18, 19); the engineering literature had already named what is achievable now (8). The organizational digital shadow is that thing: unidirectional, partial, purpose-bound, and honest about it. In the frame of the learning health system (41), it is the institutional-level analog of the patient-level learning loop, and the complement to the health digital shadow we described previously (21).

One criterion should govern its development. The test of an organizational digital shadow is not how much it sees, but whether the quality management office and the clinical teams do less manual work after it exists than before. A system that shortens the interval between deviation and detection while lengthening the time clinicians spend documenting has solved the wrong problem. Automation in quality management is justified by relief, not by visibility.

Its promise is modest to state and considerable in effect: to shorten the interval between when a certified cancer center stops meeting its own standard and when anybody notices.

## Data Availability

The original contributions presented in the study are included in the article/Supplementary Material, further inquiries can be directed to the corresponding author.
