# Supplementary material for: The organizational digital shadow of the certified cancer center—from yearly snapshots to continuous signals
Source: Front Digit Health. 2026 Oct 21;8:1955074. doi: 10.3389/fdgth.2026.1955074 (PMC13731345; doi:10.3389/fdgth.2026.1955074)
Supplement: Supplementary file 1 [file Table1.docx]

**Supplementary Material**

*The Organizational Digital Shadow of the Certified Cancer Center: From Yearly Snapshot to Continuous Signal*

Markus Knott, Markus Krebs

**Supplementary Table S1 — Management-theoretic anchors**

A Perspective constrained to three thousand words cannot develop every theoretical connection it draws on. This table states which anchors carry argumentative weight in the main text, which are invoked in passing, and what each is and is not claimed to establish. Reference numbers follow the main text.

**Table S1.** *Anchors, their role, and the limits of what each supports.*

| **Anchor** | **Core claim drawn on** | **Bearing on the ODS** | **Location** | **Role** |
| --- | --- | --- | --- | --- |
| Continuous auditing — Vasarhelyi & Halper [5]; Alles et al. [6] | Periodic audit yields a snapshot that may not reflect emerging risk; automated testing against rules can replace sampling | Supplies the operating principle of ODS-2. The certification requirement catalogue occupies the position held by internal-control assertions in financial auditing | §1, §4, §5 | Load-bearing |
| Organizational routines, ostensive and performative — Feldman & Pentland [17]; Becker & Pentland [16] | A routine has a shared, partly tacit understanding (ostensive) and the actions actually taken (performative); event data record the latter only as incomplete traces and the former not at all | The reason a shadow can never be complete, and the reason the human auditor is not made obsolete. This is the central theoretical premise of the argument | §3 [17]; §3, §6, §7, §8 [16] | Load-bearing |
| Semiotic critique of the organizational twin — Lyytinen et al. [18]; Wurm et al. [19] | A genuine twin presupposes synchronized two-way causal coupling; modelling an organization would require modelling agency, conflict and emergence | Establishes that the twin framing at the organizational level is contested by its own proponents, which is what licenses the shadow framing | §3, §7, §8 [18]; §3, §8 [19] | Load-bearing |
| Requisite variety — Ashby [30]; Viable System Model — Beer [31] | A regulator can control only as much variety as it itself commands; a representation of an organization therefore attenuates variety by selection. Viability requires a monitoring channel distinct from operations | Establishes that selective modelling is unavoidable and that the ODS is a variety attenuator by design. It does not establish that a shadow is preferable to a twin; that follows from [16]–[19], not from cybernetics | §5 | Load-bearing, with an explicit limit |
| Levers of control — Simons [32] | Control systems used diagnostically differ from those used interactively | Names the principal design risk: continuous monitoring sliding into surveillance by dashboard | §5, §6 | Load-bearing |
| Structure, process, outcome — Donabedian [27] | Care quality is assessable across three distinguishable levels | Supplies the layering of Table 1; the reflexive governance layer L4 is an addition, not part of the original framework | §5, Table 1 | Load-bearing |
| Audit culture and Goodhart — Strathern [39]; Bevan & Hood [40] | A measure ceases to be a good measure once it becomes a target; in the English target regime, real-time transparency appeared to exacerbate reactive gaming, and the authors warn that data-based surveillance substituted for site visits widens rather than narrows the audit hole | Grounds the gaming-resistance requirement, which is sharper under continuous than annual measurement, and the retention of on-site human audit | §6 | Load-bearing |
| Organizational digital twin as constructed resource — Parmar et al. [14] | Emerges through incremental construction and maintenance, not a single build | Justifies a maturity progression rather than a target architecture | §3, §5 | Supporting |
| Action-oriented process mining — Park & van der Aalst [15] | Event data can be continuously confronted with a formal model and violations turned into actions | The closest existing method analogue; the basis of the proposed demonstrator. The ODS adopts its conformance step and deliberately omits its action step | §3, §4, §5 | Method anchor |
| Dynamic capabilities — Teece [28] | Sensing, seizing and reconfiguring as a distinct organizational capacity | Characterizes what layer L4 records, as a proxy rather than a proof: the capacity to self-correct rather than a performance level | §5 (L4) | In passing |
| Double-loop learning — Argyris & Schön [29] | Single-loop learning corrects the action; double-loop learning revises the governing variables behind it | Characterizes the interpretive use of L4: asking whether the indicator is right, not only whether it is met. A recorded corrective action shows the single loop; whether the governing assumptions were revised is not shown by the record and remains a matter for human review | §5 (L4) | In passing |
| Learning health system — Institute of Medicine [41] | Care and evidence generation form a continuous loop | Positions the ODS as the institutional-level analogue of a patient-level learning loop | §8 | In passing |

**Note to Table S1**

Three anchors are invoked in passing rather than developed. This reflects the word limit of the article type. Should the article be developed at greater length, the two candidates for full development are the routine-theoretic premise, which currently carries more argumentative weight than its textual space suggests, and the levers-of-control framing, which connects the governance discussion in §6 to the design choices in §5 more tightly than the present text makes explicit.
